# Imprudence Versus Climate

**Published:** 1855-12

**Authors:** 


### IMPRUDENCE *VERSUS* CLIMATE.

"O Liberty, how many crimes are perpetrated in thy name!" exclaimed the elegant Madame Roland, as she bared her neck to the guillotine of revolutionary France. "O climate, how many deaths are charged to thy account!" may be a medical echo. Our personal experience has convinced us, that with a rational care a man may live healthfully anywhere. That men get sick and die in latitudes not their own, is the result of downright ignorance or inexcusable presumption. That some persons will die from change of climate is not denied, but it is the exception, not the rule. The great general fact is capable of the most conclusive proof, that *loss of life is not the necessary attendent of any change of latitude.* With the light which medical observation and research have thrown out, companies of men, women, and children may live in healthfulness in any climate to which they may emigrate. Persons interested, will find some most instructive and conclusive facts on this subject, in the August No. of the *Maryland Colonization Journal,* from the pen of its Editor, James Hall, M.D., who has the important advantage of writing from his own personal observation and experience.

